# Happiness and health behaviours in Chilean college students: A cross-sectional survey

**DOI:** 10.1186/1471-2458-11-443

**Published:** 2011-06-07

**Authors:** José A Piqueras, Walter Kuhne, Pablo Vera-Villarroel, Annemieke van Straten, Pim Cuijpers

**Affiliations:** 1 Departament of Health Psychology, Universidad Miguel Hernández de Elche, Elche, Spain; 2 Center for Health Promotion, Universidad de Santiago de Chile (USACH), Santiago de Chile, Chile; 3 School of Psychology, Universidad de Santiago de Chile (USACH), Santiago de Chile, Chile; 4 Department of Clinical Psychology, VU University Amsterdam, Amsterdam, The Nederlands

## Abstract

**Background:**

Happiness has been associated with a range of favourable health outcomes through two pathways: its relationship with favourable biological responses to stress and with healthy lifestyles and prudent health behaviours. There are a substantial number of cross-cultural studies about happiness, but none of them has studied the association of happiness with perceived stress and health behaviours in Latin American samples. Therefore, the aim of this study was to examine the association between general happiness and these variables in a Latin American sample.

**Methods:**

We conducted a survey to examine the status of 3461 students aged between 17 and 24 years old (mean age = 19.89; SD = 1.73) who attended University of Santiago de Chile during 2009. The healthy behaviours indexes assessed were the frequency of daily physical exercise, fruits/vegetables intake, breakfast and lunch intake, smoking, alcohol and other drugs consumption. We also included the assessment of perceived stress and Body Mass Index. All of them were evaluated using a self-report questionnaire.

**Results:**

The univariate and multivariate binary logistic regression analyses showed that being female and younger was related to a higher happiness, as well as that people self-reporting daily physical activity, having lunch and fruits and vegetables each day had a higher likelihood (OR between 1.33 and 1.40) of being classified as "very happy". Those who informed felt stressed in normal circumstances and during tests situations showed a lower likelihood (0.73 and 0.82, respectively) of being considered "very happy". Regarding drug consumption, taking tranquilizers under prescription was negative related to "subjective happiness" (OR = 0.62), whereas smoking was positive associated (OR = 1.20).

**Conclusions:**

The findings of this study mainly support the relationship between happiness and health outcomes through the two pathways previously mentioned. They also underscore the importance of that some healthy behaviours and person's cognitive appraisal of stress are integrated into their lifestyle for college students. Additionally, highlight the importance of taking into account these variables in the design of strategies to promote health education in university setting.

## Background

In recent years, research on positive psychology has emerged highlighting the role of positive psychological variables in making life more successful, improving human functioning, and increasing happiness [1]. Broadly, the literature has distinguished between two dimensions of "wellbeing" or "positive mental health" [2,3]: hedonic or subjective well-being, which emphasizes constructs such as positive feelings, positive affect, subjective well-being, life satisfaction or happiness; and eudemonic or psychological well-being tradition, which accentuates positive psychological functioning and human development (i.e., engagement, fulfilment, sense of meaning, social wellbeing).

Among constructs related to positive well-being, research into happiness has increased considerably over the past forty years. Some of the most widely accepted definitions of happiness [1,4-6] agree that subjective well-being is defined as the evaluative reaction of a person to his/her life and it can be divided into a cognitive component (cognitive evaluation of life satisfaction) and an affective component (emotional aspects of the construct, such as happiness). Therefore, subjective positive well-being is a broad category of phenomena, whereas happiness specifically represents the affective evaluation of one's life, although they are often regarded as synonymous [7].

Broadly, there is accumulating evidence (cross-sectional, longitudinal and experimental research) that positive well-being is associated with many resources valued by society, such as healthy behaviours, lower delinquent activity, higher incomes, superior mental health, a higher education, a long life, better performance ratings at work, an improved social and personal functioning, lower heavy Internet and game use, etc. [7-15]. In short, the results reveal that happiness has multiple benefits, being associated with and precedes numerous successful outcomes, as well as behaviours paralleling success. Furthermore, the evidence suggests that positive affect--the hallmark of well-being--may be the cause of many of the desirable characteristics, resources, and successes correlated with happiness [7].

Specifically, the direct effect of well-being on future health has been proposed to be mediated through two pathways: the association between positive psychological states, such as happiness, and a lower perceived stress and/or favourable biological responses to stress (including low cortisol levels, reduced cardiovascular disease risk, faster cardiovascular stress recovery, reduced inflammation, and resilience to infection) and the relationship with healthy lifestyles and prudent health behaviours that reduce long-term risk of disease development [16].

As regards stress, in general empirical evidence suggests that there are marked associations between positive psychological states, such as happiness, and perceived stress, indicating that there was an inverse relationship between these variables [14]. At the biological level, cortisol output has been consistently shown to be lower (or at least a more flexible response) among individuals reporting positive affect, and favourable associations with heart rate, blood pressure, and inflammatory markers such as interleukin-6 have also been described [17].

Regarding healthy lifestyles and prudent health behaviours, previous studies have found that there is a significant association between happiness and health outcomes, such as exercising regularly or higher levels of physical exercise, not smoking or less cigarette use, less alcohol intake, higher sleep quality and quantity, and prudent diet [18-21]. Therefore, happy individuals are less likely to engage in a variety of harmful and unhealthy behaviours, including smoking, unhealthy eating, and abuse of drugs and alcohol [22]. Thus, positive affect might benefit health by indirect relations to health promoting activities.

Results of studies concerning the association between obesity and happiness or well-being are conflicting. Some of them did not find evidence that increased weight impairs happiness [19,23]. However, a study found that a significant U-shaped trend in the association between body mass index categories (underweight, normal, overweight and obesity) and depression, which could be considered as the opposite extreme of happiness [24].

In spite of these data, following Grant et al. [16] or Steptoe et al. [11] the association between positive affect and healthy behaviour choices are quite mixed and some results have been inconsistent [19,25-27]. Even less is known about associations between well-being and other health behaviours such as dietary choice.

Furthermore, despite there is a substantial number of cross-cultural studies about well-being and happiness [16,28,29], none of them has studied the relationship between happiness and health outcomes in Latin American college samples previously.

Therefore the main aim of this study was to confirm associations between subjective happiness and perceived stress and the relationship between happiness and healthy lifestyles and prudent health behaviours, such as physical activity, smoking, and alcohol consumption, and test relationships with other behaviours, specifically with three aspects of food choice (fruit/vegetables, breakfast and lunch intake) and with body index mass categories following the World Health Organization recommendations.

We hypothesized that happiness would be positively associated with lower scores on perceived stress, supporting the idea that favourable response to stress (including biological correlates and perceived stress) is related to positive psychological states. We also expected a significant relationship between happiness and some healthy behaviours (a better diet quality, daily exercise and the lack of any drug taking). Both outcomes would give partial support to the idea of that mediation of happiness in reducing long-term risk of disease development through both pathways also appear in Latin American samples.

## Methods

### Participants

The data analyzed for this study were taken from the Quality of Life and Health Behaviours Survey, a cross-sectional questionnaire survey of university students administered in the University of Santiago de Chile in 2009. Respondents were enrolled on a variety of programs, including Administration and Economy, Science, Medical Sciences, Humanities, Engineering, Technology, Chemistry and Biology, Architecture and Bachelor program. They belonged to 166 of the 346 municipalities (comunas) in which Chile is divided, so that the socio-economic status of the sample can be considered broad and representative of universities from this country.

### Study sample

The sample consisted of 3461 students, 1866 (53.90%) males and 1595 (46.10%) females, aged between 17 and 24 years old (mean age = 19.89; SD = 1.73).

The questionnaires were typically administered at the end of classes. The inclusion criterion was to belong to any program of the University of Santiago de Chile. The application was made by graduate students who had been trained on the tool. Students were informed that the survey measured activities relevant to health. We requested them written consent after informing them that completion of the survey was voluntary, anonymous and confidential. Those questionnaires incomplete or inadequately answered were eliminated.

### Measures

The Quality of Life and Health Behaviours Survey included general information, such as age, gender, study program, body weight and height, and a range of measures of perceived stress and health risk behaviours, such as frequency of drug taking, physical activity, eating vegetables/fruits, etc. The Subjective Happiness Scale (SHS) were also organized in the questionnaire.

#### Evaluation of happiness

- Subjective Happiness was assessed with the Spanish version of the SHS [5]. It consists of four items rated on a 7-point Likert scale, where individuals had to indicate whether they agreed or disagreed with statements. Items contents are: "1. In general, I consider myself a very happy person"; "2. Compared to most of my peers, I consider myself more happy"; "3. Some people are generally very happy. They enjoy life regardless of what is going on, getting the most out of everything. To what extent does this characterization describe you?'' and "4. Some people are generally not very happy. Although they are not depressed, the never seem as happy as they might be. To what extent does this characterization describe you?" (item 4 is reverse coded). A single SHS score is computed by taking the mean of responses to the four items, so scores can range from 1 to 7. Previous studies have shown that the SHS has good-to-excellent internal consistency (0.79-0.94), test-retest reliability (0.72), and convergent and discriminant validity across different languages, countries and cultures, including Spanish [5,29-33]. This approach to happiness focuses on a method to capture the global and subjective qualities of happiness and attempts to allow the individual to give an overall assessment of the extent to which he or she is a happy person. Thus, it identifies a relatively stable characteristic of happiness separate from life experiences. It resulted in one of the most used self-report scales to measure this construct. Based on the 75^th ^percentile score (scores of 6 or above on the 7-point Likert scale), respondent were categorized as either non happy (coded as 0) or happy (coded as 1).

#### Evaluation of perceived stress

- Perceived stress was measured using two items: "How stressed are you during an ordinary week in the university?" and "How stressed are you during exam periods and ends of semester?" Responses were rated on a scale from 1 to 4, ranging from "not stressed at all" to "very stressed". The scores were become into a binary variable based on the 75^th ^percentile score, with 0 meaning few or not stressed regarding the perceived stress in ordinary days (scores of 3 or above) and for the test situations-related stress (scores of 4). These items were selected from the questionnaire developed by the National Council for Narcotics Control (Consejo Nacional para el Control de Estupefacientes, CONACE) for the National Study of Drugs in General Population of Chile 2008 [34].

#### Evaluation of health behaviours

The health behaviours included in this study were assessed using single items:

- *Consumption of legal and illegal drugs*. The items were selected from the same questionnaire developed by the CONACE for the National Study of Drugs in General Population of Chile 2008 [34]. It attempts to measure consumption of legal and illegal drugs by means of the following question: When was the last time you tried any of these drugs? Responders can rated on a scale from 1 to 4 ("1 = Never", "2 = More than 1 year ago", "3 = More than a month, but less than one year" and "4 = During the past 30 days". They are categorized as either non-takers (code 0 = scores 1 or 2) or takers (coded as 1 = scores 3 or 4), following the 75^th ^percentile score.

- *Nutrition aspects and psychical activity *were assessed using five options from "Never = 1" to "Daily = 5" to evaluate the frequency of breakfast, lunch, eating fruit and vegetables, and doing exercise. The healthy option (coded as 1) was having breakfast daily, having lunch daily and eating fruits and vegetables daily or almost daily, and doing psychical activity daily or almost daily, respectively. This decision was assumed following the 75^th ^percentile score as the cut-off point.

- *The nutritional status *was calculated from self-reported height and weight. We followed criteria of the World Health Organization (World Health Organization, WHO) [35] for BMI categories: underweight (BMI ≤ 18.49 kg m-2), normal weight (BMI = 18.50 to 24.90 kg m-2), overweight (BMI = 25.00 to 29.90 kg m-2) and obesity (BMI ≥ 30.00 kg m-2). Underweight was the reference category (coded as 0).

### Data Analysis

The distribution of happiness ratings across gender, age, perceived stress and health behaviours was analyzed using descriptive statistics and presented as mean and standard deviation (SD) for happiness (dependent variable) and as percentages for independent categorical variables. We settled the differences across these variables on happiness by Student's t-test and ANOVAs'. Cohen's d index (d) for t-tests and eta-squared (η^2^) for ANOVAS was included for valuing the effect size (ES). The interpretation of Cohen's d index is as follows: small (.20 - .49), medium (.51 - .79) and large effect sizes (d ≥ .80), whereas eta-squared is interpreted as the ratio of variance explained in the dependent variable by a predictor.

To establish the association between happiness and each independent variable, first of all we conducted univariate binary logistic regression analysis with happiness as the dependent variable and each variable as the independent one. Most variables were previously dichotomized to the values 0 or 1 based on the 75^th ^percentile value or highest quartile (cut-off point) in order to facilitate odds ratio computations and interpretation. Then, all predictive factors that had a p-value less than 0.20 in univariate analysis were entered in multivariate binary logistic regression. We used the method "forward LR selection of variables". With that method, variables with the highest predictive value are added stepwise till estimates change by less than 0.001. Thus, not all predictors are entered in the analysis. Nine steps were carried out. The odds of healthy behaviour and perceived stress for each level of happiness were computed, with "non happy" as the reference category. Statistically significant results with a significance level ≤ 0.05 were considered. We used SPSS version 18.0 for Windows on its processing and statistical analysis.

## Results

### Socio-demographical features

The distribution by age in relation to gender is showed in table 1. A significant association was found between age and gender (χ^2 ^= 21.90, df = 7, p = 0.003). The main socio-demographical attributes for the total group are listed in table 2. Of the respondents 1595 (46.10%) were female and 1866 (53.90%) were male with a mean age of 19.89 (SD = 1.73, ranging from 17 to 24 years). Although the distribution by age was homogeneous, females were older, with a mean age of 19.98 ± 1.73 years by comparison with 19.81 ± 1.73 years in males (t = -2.94, df = 3459, p = 0.003, d = -0.10).

**Table 1:** Age × gender distribution of college sample in Santiago de Chile, Chile, 2009 (n = 3461)

	Gender		
**Age**	**Male**	**Female**	**Total**

**(years)**	**n (%)**	**n (%)**	**n (%)**

17	10 (0.30)	17 (0.50)	27 (0.80)

18	525 (15.20)	363 (10.50)	888 (25.60)

19	443 (12.80)	374 (10.80)	817 (23.60)

20	318 (9.20)	276 (8.00)	594 (17.20)

21	238 (6.90)	237 (6.80)	475 (13.70)

22	137 (4.00)	156 (4.50)	293 (8.50)

23	129 (3.70)	117 (3.40)	246 (7.10)

24	66 (1.90)	55 (1.60)	121 (3.50)

Total	1866 (53.90)	1595 (46.10)	3461 (100)

**Table 2:** Associations of happiness with demographic variables and with perceived stress and health behaviours

Variables	Variable categories	% of total sample	Happiness score (mean; SD)	p-value
Demographics				

Gender	Female	46.10	5.25; 1.10	0.24
	Male	53.90	5.21; 1.10	

Age	17-19	50.00	5.27; 1.10	0.02
	20-24	50.00	5.19; 1.09	

Nutritional status (WHO)				

Body Mass Index (BMI)	Underweight	3.40	5.17; 1.27	0.09
	Normal weight	77.60	5.25; 1.08	
	Overweight	16.90	5.22; 1.12	
	Obese	2.10	4.93; 1.13	
	No obese	97.90	5,25; 1.10	0.02
	Obese	2.10	4,93; 1,13	

Daily breakfast	No always	50.50	5.15; 1.12	0.00
	Always	49.50	5.32; 1.07	

Daily lunch	No always	36.00	5.04; 1.17	0.00
	Always	64.00	5.34; 1.04	

Daily fruits and vegetables	No always	44.50	5.10; 1.11	0.00
	Always	55.50	5.34; 1.07	

Physical activity	No always	71.00	5.17; 1.11	0.00
	Always	29.00	5.39; 1.06	

Stress				

Ordinary circumstances	No stressed	37.80	5.42; 1.04	0.00
	Stressed	62.20	5.11; 1.12	

Exam/test periods	No stressed	33.20	5.44; 1.01	0.00
	Stressed	66.80	5.13; 1.12	

Drug use				

Tobacco	No	55.30	5.22; 1.08	0.46
	Yes	44.70	5.25; 1.10	

Alcohol	No	16.80	5.21; 1.12	0.63
	Yes	83.20	5.24; 1.09	

Prescriptive tranquillizers	No	89.80	5.27; 1.07	0.00
	Yes	10.20	4.90; 1.21	

Modafinil (i.e., Mentix)	No	95.30	5.24; 1.09	0.32
	Yes	4.70	5.15; 1.13	

Inhalants	No	98.70	5.23; 1.09	0.55
	Yes	1.30	5.33; 1.04	

Marihuana	No	75.40	5.21; 1.10	0.04
	Yes	24.60	5.30; 1.06	

Cocaine	No	98.20	5.23; 1.09	0.93
	Yes	1.80	5.25; 1.13	

Unprescriptive tranquilizers	No	96.00	5.25; 1.08	0.00
	Yes	4.00	4.88; 1.30	

Unprescriptive analgesics	No	93.50	5.25; 1.08	0.01
	Yes	6.50	5.06; 1.20	

Unprescriptive amphetamines	No	98.40	5.24; 1.09	0.05
	Yes	1.60	4.95; 1.18	

Other drugs < 1% (heroine, hallucinogens, ecstasy)	No	98.50	5.23; 1.09	0.12
	Yes	1.50	5.47; 1.06	

Number of "illegal" drugs (range = 1-11)	0	65.90	5.25; 1.09	0.76
	1	26.00	5.23; 1.08	
	2	6.00	5.18; 1.17	
	3	2.10	5.14; 1.15	

* p-values < 0.05 using students-T and Anovas-F.

### Distribution of predictive variables and comparisons on happiness across these variables

The differences of happiness ratings across gender, age, perceived stress and health behaviours are shown in table 2. There were significant differences between happiness score across age group (t = 2.35, df = 3474, p = 0.019, d = 0.08), obesity status (t = 2.34, df = 3230, p = 0.019, d = 0.08), daily breakfast (t = -4.52, df = 3464, p = 0.000, d = - 0.15), daily lunch (t = -7.71, df = 3463, p = 0.000, d = -0.26), daily fruits and vegetables (t = -6.44, df = 3464, p = 0.000, d = - 0.21), daily psychical exercise (t = -5.53, df = 3461, p = 0.000. d = - 0.19), stress in ordinary periods (t = 8.08, df = 3462, p = 0.000, d = 0.27), stress in test periods (t = 8.01, df = 3463, p = 0.000, d = 0.27), prescriptive tranquilizers (t = 6.00, df = 3445, p = 0.000, d = 0.20), marihuana (t = -2.09, df = 3442, p = 0.036, d = 0.13), unprescriptive tranquilizers (t = 3.85, df = 3448, p = 0.000, d = 0.13) and unprescriptive analgesics (t = 2.47, df = 3446, p = 0.014, d = 0.08). The rest of the comparisons were not significant (see table 2). Thus, although effect sizes were small, higher scores on happiness were found among those participants who were younger, non-obese; had daily breakfast, lunch and/or fruits and vegetables; did daily physical activity; felt non-stressed; did not take prescriptive tranquilizers, neither unprescriptive tranquilizers nor analgesics, but acknowledged having taken marihuana recently.

Further analysis of these findings found that there were not significant differences between happiness score across municipalities (comunas) [F(162,3244) = 0.86, p = 0.89, η^2 ^= 0.04].

### Happiness

The mean total score on "subjective happiness" was 5.23 ± 1.10 (range = 1-7). Skewness and kurtosis were -0.59 and 0.12, respectively. Kolmogorov-Smirnov's test showed that happiness total score distribution was non normal (p <.001).

Overall, 30.80% of respondents rated themselves as very happy (scores of 6 or above on a 7-point Likert scale). So, approximately one third of participants in this study reported being happy. The percentage of "happy" across variables is shown in table 3.

**Table 3:** Univariate and multivariate odds ratios for the relationship of happiness with demographic characteristics, perceived stress and health behaviour

Variables	Variable categories	% happy	Unadjusted (univariate)		Adjusted (multivariate)	
			**Odds ratio (95% CI)**	***p***	Odds ratio (95% CI)*	***p***

Demographics						

Gender	Male (= ref)	29.80				
	Female	31.90	1.10 (0.96-1.28)	0.18	1.32 (1.12-1.55)	0.00

Age	17-19 (= ref)	32.70				
	20-24	28.80	0.83 (0.72-0.96)	0.01	0.84 (0.72-0.98)	0.03

Nutrition						

Body Mass Index (BMI)	Underweight(= ref)	33.30		0.14		
	Normal weight	31.30	0.91 (0.61-1.37)	0.06		
	Overweight	31.40	0.91 (0.59-1.41)	0.69		
	Obese	17.90	0.44 (0.21-0.91)	0.03		
	No obese	31.40				
	Obese	17.90	0.48 (0.25-0.89)	0.02		

Daily breakfast	No always (= ref)	27.30				
	Always	34.10	1.38 (1.19-1.59)	0.00		

Daily lunch	No always (= ref)	25.70				
	Always	33.50	1.46 (1.25-1.70)	0.00	1.40 (1.18-1.66)	0.00

Daily fruits and vegetables	No always (= ref)	26.20				
	Always	34.30	1.47 (1.27-1.70)	0.00	1.34 (1.14-1.58)	0.00

Daily physical activity	No always (= ref)	29.00				
	Always	34.80	1.30 (1.12-1.53)	0.00	1.33 (1.12-1.58)	0.00

Stress						

Ordinary circumstances	No (= ref)	36.50				
	Yes	27.20	0.65 (0.56-0.75)	0.00	0.73 (0.61-0.87)	0.00

Exam/test periods	No (= ref)	36.30				
	Yes	27.90	0.68 (0.58-0.79)	0.00	0.82 (0.68-0.99)	0.04

Drug use						

Tobacco	No (= ref)	29.60				
	Yes	32.10	1.12 (0.97-1.30)	0.11	1.20 (1.03-1.40)	0.02

Alcohol	No (= ref)	29.60				
	Yes	30.90	1.07 (0.88-1.30)	0.52		

Prescriptive tranquillizers	No (= ref)	31.70				
	Yes	22.40	0.62 (0.48-0.80)	0.00	0.62 (0.50-0.85)	0.00

Modafinil (i.e., Mentix)	No (= ref)	30.90				
	Yes	30.10	0.96 (0.68-1.36)	0.83		

Inhalants	No (= ref)	30.80				
	Yes	26.10	.79 (.41-1.53)	0.49		

Marihuana	No (= ref)	30.20				
	Yes	32.50	1.12 (0.95-1.32)	0.19		

Cocaine	No (= ref)	30.80				
	Yes	30.60	0.99 (0.58-1.71)	0.98		

Unprescriptive tranquilizers	No (= ref)	31.10				
	Yes	21.90	0.62 (0.41-0.94)	0.02		

Unprescriptive analgesics	No (= ref)	31.10				
	Yes	26.50	0.80 (0.59-1.08)	0.15		

Unprescriptive amphetamines	No (= ref)	30.90				
	Yes	20.40	0.57 (0.29-1.11)	0.10		

Other drugs < 1% (heroine, hallucinogens, ecstasy)	No (= ref)	30.50				
	Yes	30.00	1.52 (0.86-2.68)	0.15		

Number of "illegal" drugs (range = 1-11)	0	31.10				
	1	30.20	0.96 (0.81-1.13)	0.22		
	2	31.90	1.03 (0.76-1.41)	0.30		
	3	24.30	0.71 (0.41-1.23)	0.23		

* Method: Forward LR (they were just entered significant variables; variables not included in the final model were the following: age, physical activity, marihuana, tranquilizers without prescription, and analgesics without prescription.

### Happiness and socio-demographical characteristics

Across the complete sample, those with higher subjective happiness were more likely to be younger than those with lower happiness (OR = 0.83, p = 0.01). This association was still present when we controlled for the interrelations between the variables in the multivariate analyses (OR = 0.84, p = 0.03).

The gender did not emerge as a significant predictor in the univariate analysis (p = 0.18), but when it was entered into the multivariate analysis, it emerged as a significant independent predictor for happiness (OR = 1.32, p = 0.001), being more common among females to inform high happiness.

### Happiness and perceived stress

The proportion of respondents who reported feeling very happy was more common among those participants who reported feeling few or not stressed as well in ordinary as in test/exam periods (OR = 0.65 and 0.68, respectively, p = 0.000). The adjusted (multivariate) ORs were also significant (0.73 and 0.82 with p = 0.001 and p = 0.041, respectively). The association between happiness and perceived stress is shown in table 3.

### Happiness and health behaviours

#### Nutrition

A moderate association between unhappiness and obesity emerged (OR = 0.48, p = 0.020). However, this effect was not significant in the multivariate analysis.

About one third of the happy respondents had breakfast, lunch and fruit and vegetables almost always or always (daily). Happiness was positively associated with all these nutritional variables (ORs between 1.38 and 1.47, p = 0.000). Adjusted ORs showed the same significant association only between happiness and daily lunch and fruit/vegetables intake (Adjusted ORs 1.40 and 1.34, respectively, p = 0.000).

#### Physical activity

A moderate but significant association with happiness emerged (OR = 1.30, p = 0.001), showing that daily and regular exercise is more frequent among happier participants. This association was still present when the interrelations between the variables were controlled in the multivariate analysis (Adjusted OR = 1.33, p = 0.001).

#### Drug intake

Univariate analysis showed that happiness was associated with either prescriptive or unprescriptive tranquilizers takers (OR = 0.62 and 0.62, p = 0.000 and p = 0.020, respectively). In the multivariate analysis, only users of tranquilizers under prescription was longer statistically associated with subjective happiness (Adjusted OR = 0.620, p = 0.001), indicating that unprescriptive tranquilizers differences were mediated by other variables. Unexpectedly, smoking cigarettes emerged as a significant predictor of happiness in the multivariate analysis (Adjusted OR = 1.202, p = 0.021), indicating that its effect were mediated by other variables.

## Discussion

The main objective of the study was to confirm associations between subjective happiness (one of the construct related to positive psychological states and well-being) and favourable health outcomes in a Latin American sample. Traditionally, it has been purposed that this relationship takes place through two pathways: its relationship with favourable biological responses to stress and its connection with healthy lifestyles and prudent health behaviours. On the one hand, we pretended to give support to the relationship between happiness and lower scores on perceived stress, as a correlate of favourable psychobiological responses to stress. On the other hand, we tried to find some evidence of the association of happiness with some health behaviours, as an expression of healthy lifestyles and prudent health behaviours.

A substantial number of young adults in this study reported very high happiness, with 30.80% of the total sample saying that they were very happy with their lives (scores of 6 or above in a 7-point Likert scale). Even more, if we considered people over the mean score, the rate was higher, approximately 50%. This data are consistent with those indicating that 23% (range = 12-27%) of young adults reports being very satisfied with their life as a whole (very positive well-being) across culture [16] or other study showing an average level of "very happy" people of 27.5% (range = 8-47%). In general, all these studies found that subjective well-being or happiness is higher in Western countries, followed by countries from Eastern Europe and finally Asian countries [16,29,36].

Our results indicated that being very happy was more common among female. Although gender effect has previously presented some ambiguity, in general, it appears that women tend to report higher happiness levels than men [16,37]. Anyway, it seems to be evidence of paradoxical women's declining relative wellbeing across demographic group and industrialized countries over recent decades in comparison with the opposite effect among men [38]. Furthermore, some studies display that there is a specific difference in the gender distribution of well-being across world regions, with women reporting greater levels of well-being on average in Asian countries, but lower levels than men in the Western countries [16]. Anyway, genetic studies do not find any age effect [39,40].

Referring to the relationship between age and happiness, we found that happiness was higher in the younger group. This data is consistent with those by Dear et al. [37] that found that life satisfaction was higher in young adults than in the middle -aged or elderly or Bartels et al. [39] who reported a small but significant negative effect of age on mean levels of subjective well-being. Although some studies have found the opposite age effect, highlighting that aging is a preventative factor of depressive state and/or felling of unhappiness [19], or have not showed any age effect [40], this apparent contradiction has been clarified recently. Some studies have proved that there are non-linear effects, but well-being is U-shaped over the life cycle. In general, these studies show that lower levels of happiness are among the 35 and 62 years of age -middle age- across gender and countries [41]. Furthermore, this work confirmed this result for a large, heterogeneous sample of countries (72 developed and developing countries), taking into account potential cohort effects. This finding is also coherent with our data, corresponding with some point of the increasing left tail of the U-shaped distribution.

The hypothesis related to the association between happiness and perceived stress was largely confirmed (in both univariate and multivariate analyses), indicating that participants who perceived higher levels of stress in ordinary circumstances and during tests situations reported being less happy than those with lower levels of stress. These results are in line with previous evidence showing that there is an inverse relationship between happiness with perceived stress by means of self-reported measures [14,17]. So, Schiffrin et al. [14] found that both self-reported variables were inverse associated by means of a correlational study with college students, whereas Mikolajczak et al. [17] also found the same effect plus a relationship between subjective happiness and a biological marker of psychological and physical health status, the cortisol awakening response flexibility. This study is consistent with previous studies [14] suggesting that one of the main practical implications of this finding is that interventions designed to increase happiness might benefit from the inclusion of activities to manage and cope with stress and that this sort of interventions should also utilize state measures of happiness that are sensitive to increases in happiness that may occur as a result of the intervention.

The second main hypothesis of the study was partially confirmed, in that happiness was positively associated with most of the prudent health behaviours except alcohol consumption, and other drug uses. Effects were positive and significant, but moderate for daily breakfast, daily lunch, daily fruit and vegetables intake and daily physical activity, as well as they were negative for prescriptive and unprescriptive tranquilizers intake and obesity. When the calculations were adjusted by means of the multivariated analysis, obesity, daily breakfast and unprescriptive tranquilizers intake were no longer statistically associated with subjective happiness, but smoking emerged as a new significant predictor of happiness to be added to the previous mentioned variables. In general, our results therefore add to the limited data currently available relating well-being and happiness with prudent health behaviour in Latin American countries.

Overall, our results are consistent with other studies that pointed out that life satisfaction is positively associated with most of the prudent health behaviours across culturally diverse countries, with effects strong for psychical exercise, intermediate for fruit intake and lower but significant for cigarette smoking and dietary fat avoidance [16]. In addition to this, other studies have also found an association of high self-rated health with more physically active, more sleep, less likely to be overweight, lower scores on loneliness, shyness and hopelessness, and higher on self-rated happiness [12] and between positive lifestyle changes such as increasing physical activity levels and increase in fruit and vegetable consumption and positive changes in mental health (peacefulness and happiness) [42].

Regarding physical activity, they are also coherent with a study showing that exercise participation is associated with higher levels of life satisfaction and happiness, and that both variables appeared to be mediated by genetic factors [21] or with another one highlighting a inversely association between jogging and other types of psychical activity in leisure time with stress and life dissatisfaction [43]. Most of these studies recommend that increased well-being should be a key argument in future campaigns for increased leisure-time physical activity.

Previous research relating dietary quality with positive well-being has been inconsistent, at least in Western countries, regarding daily breakfast and snacks intake [16,19]. Other previous works reported a significant relationship with fruits and vegetables and limiting fat intake [16,42]. Our findings give a stronger support to this relationship related to daily intake of lunch and fruit and vegetables, and a significant, but lower support to the association with daily breakfast intake. Therefore, our findings are consistent with these previous studies.

Regarding obesity, our data showed a low but significantly positive relationships between obesity and a lower happiness. Although previous studies indicated contradictory outcomes regarding the association between obesity or BMI categories and happiness or well-being [19,23], however, our outcome is consistent with those by de Wit et al. [24] showing a significant U-shaped trend in the association between body mass index categories (underweight, normal, overweight and obesity) and depression, which could be considered as the opposite extreme of happiness. Probably, the lack of agreement could be made clear because of most studies focused on linear- (positive, negative) or no trends in the association between obesity and depression/happiness, whereas a u-shaped association is a better explanation.

Our results showed that happiness was moderately and negatively related to prescriptive tranquilizers intake, and lower but also significantly with unprescriptive tranquilizers intake. This finding is in line with the controversy about the use of the psychotropic drugs labelled as happiness pills or "happy pills" during the last half 60 years. In this sense, some authors have highlighted that "happiness pills" has become a "national nightmare" [44]; the problem of iatrogenic addiction in the age of happiness pills as 'Botox' for the mind [45] or the unhappy saga of happy pills [46].

Surprisingly, we also found a low positive association between happiness and smoking by means of the multivariate analysis, whereas we did not find any relationship between happiness and other drugs consumptions, such as alcohol intake, marihuana consumption, etc. The results about the association between positive affect and healthy behaviour have been quite have been inconsistent [19,25-27]. So, on the one hand, some studies have found a relationship of well-being with no smoking or less cigarette use [11,16,22,47]; with less alcohol intake [27] and with no smoking nor abuse of drugs or alcohol [22]. On the other hand, other works did not find this relationship between neither smoking nor alcohol intake and happiness [19]. Even, other studies found a positive relationship between alcohol intake and higher well-being among university students [26] or a U-shaped relationship between well-being and alcohol consumption, whereby well-being was lower both in abstainers and in heavy users [37]. Thus, positive affect might benefit health by indirect relations to health promoting activities, although the relationship seems to be more complex than expected. In example, a tentative explanation for the inconsistency about the relationship between smoking and well-being has been that smoking might partly be a consequence of negative affective states, while stopping smoking or reducing cigarette consumption leads to enhanced well-being and happiness [48].

To sum up, our findings are mainly consistent with previous studies, indicating that healthy lifestyles and prudent health behaviours are positively associated with happiness. Furthermore, studies have pointed out that both genes and environment play important roles in the associations between well-being and health [49].

This study has a number of strength, including a large homogeneous sample, uniform measures of health behaviours, a standard assessment of happiness and the novelty of including a Latin American sample. There are also several limitations. This study was cross-sectional, so causal relationships cannot be drawn. The study was carried out with students from a public university of one country from Latin America, and inclusion of other centres could have resulted in different effects. The association between happiness and food might be moderated by income. Despite the fact that participants belonged to a broad variety of municipalities from Chile with different level of incomes following the Chilean National Institute of Statistics, there was no data on participants' income. Furthermore, our university students sample are not representative of Chilean young adults in general, and the rate of happiness and health behaviours may be different in other sector of the population. Students were tested here by means of self-report items and scales, and more refined assessments with objective verification would have been desirable.

## Conclusions

Nevertheless, the results add to the literature in documenting associations between positive well-being/happiness and a range of behaviours and emotional responses relevant to health in a different cultural group. The findings of this study are consistent with the notion that health behaviours and perceived stress account in part for the relationship between positive psychological states and good health, providing support to the double pathway in which well-being seems to have an effect on health outcomes. It also underscore the importance of that some healthy behaviours and person's cognitive appraisal of stress are integrated into their lifestyle for college students. Additionally, highlight the importance of taking into account these variables in the design of strategies to promote health education in university setting. Finally, our findings pointed out the extent of happiness in an emerging country such as Chile, which is comparable to that of western countries belonging to the so-called "western culture" (thus despite economic and other cultural differences).

## Competing interests

The authors declare that they have no competing interests.

## Authors' contributions

All authors contributed equally in the design of the study. JAP oversaw all aspects of its implementation, performed the statistical analysis and prepared the manuscript. WK and PVV acquired funding and permissions for the research, collected the data and assisted in drafting of the manuscript. AVS and PC gave statistical expertise, assisted in interpretation of data analysis and revised the manuscript for intellectual content. All authors read and approved the final manuscript.

## Pre-publication history

The pre-publication history for this paper can be accessed here:

http://www.biomedcentral.com/1471-2458/11/443/prepub
